# TNF-α Promoter Polymorphisms Predict the Response to Etanercept More Powerfully than that to Infliximab/Adalimumab in Spondyloarthritis

**DOI:** 10.1038/srep32202

**Published:** 2016-08-31

**Authors:** Jing Liu, Zheng Dong, Qi Zhu, Dongyi He, Yanyun Ma, Aiping Du, Fan He, Dongbao Zhao, Xia Xu, Hui Zhang, Li jin, Jiucun Wang

**Affiliations:** 1 State Key Laboratory of Genetic Engineering and Ministry of Education (MOE) Key Laboratory of Contemporary Anthropology, Collaborative Innovation Center for Genetics and Development, School of Life Sciences, Fudan University, Shanghai, China; 2 Guanghua Integrative Medicine Hospital, Shanghai, China.; 3 Institute of Arthritis Research, Shanghai Academy of Chinese Medical Sciences, Shanghai, China; 4 Department of Rheumatology and Immunology, Changhai Hospital, affiliated to second military medical university, Shanghai, China; 5 Institute of Rheumatology, Immunology and Allergy, Fudan University, Shanghai, China

## Abstract

While previous studies have researched in association analyses between TNFα promoter polymorphisms and responses to TNF blockers in spondyloarthritis patients, their results were conflicting. Therefore, we aimed to determine whether TNFα promoter polymorphisms could predict response to TNF blockers and find the source of heterogeneity. Data were extracted and analyzed from published articles and combined with our unpublished data. We found that the greatest potential sources of heterogeneity in the results were gender ratio, disease type, continents, and TNF blockers. Then Stratification analysis showed that the TNFα −308 G allele and the −238 G allele predicted a good response to TNF blockers (OR = 2.64 [1.48–4.73]; 2.52 [1.46–4.37]). However, G alleles of TNFα −308 and −238 could predict the response to etanercept (OR = 4.02 [2.24–7.23]; 5.17 [2.29–11.67]) much more powerfully than the response to infiliximab/adalimumab (OR = 1.68 [1.02–2.78]; 1.28 [0.57–2.86]). TNFα −857 could not predict the response in either subgroup. Cumulative meta-analysis performed in ankylosing spondylitis patients presented the odds ratio decreased with stricter response criteria. In conclusion, TNFα −308 A/G and −238 A/G are more powerful to predict the response to Etanercept and it is dependent on the criteria of response.

Spondyloarthritis (SpA) is a group of inflammatory diseases comprising five subgroups: ankylosing spondylitis (AS), psoriatic spondyloarthritis (PsA/Ps), reactive spondyloarthritis, spondyloarthritis associated with inflammatory bowel disease (IBD/CD), and undifferentiated spondyloarthritis. They share common clinical and genetic features^1^. Clinical features include inflammation of the axial skeleton, asymmetrical peripheral oligoarthritis (predominantly of the lower limbs), and disorders of specific organs, such as anterior uveitis and psoriasis^2^. The five SpA subgroups are genetically linked by the MHC class I molecule HLA-B27^1^.

Tumor necrosis factor-a (TNFα) is an inflammatory cytokine that has been implicated in the pathogenesis of spondyloarthritis. TNFα inhibits collagen synthesis in osteoblasts and stimulates the synthesis of proteolytic enzymes such as plasminogen activators and matrix metalloproteinases. In addition, activation of osteoclast production by TNFα promotes bone resorption and joint damage, which results in disease progression of AS^3^. In synovial fibroblasts, TNFα upregulates the expression of Angiopoietin-1 (Ang-1), which regulates angiogenesis through activation of the transcription factor AP-1 and the NF-κB signal transduction pathway^4^. Angiogenesis occurs together with chronic inflammation, and both processes lead to increases in cellular infiltration and proliferation, regulatory growth factors, and cytokines^5^.

In recent years, TNF-blocking agents have been used widely in the treatment of SpA, especially in AS^6^,^7^. TNFα antagonists comprise primarily two main types of agents: monoclonal antibodies (e.g., adalimumab (ADA) and infliximab (INF)) and soluble receptors (e.g., TNFR:Fc and etanercept (ETA)). Although anti-TNFα agents can effectively stop or slow disease progression in some patients^8^, other patients do not respond to treatment with these agents. Previous studies have shown that 25% of SpA patients had no response to TNFR:Fc agents, while 21% had no response to anti-TNFα monoclonal antibodies^9^,^10^,^11^.

The response of SpA patients to TNFα blockade is associated with several SNPs in the TNFα promoter region. Among these, the polymorphisms TNFα −308 A/G, −238 A/G, and −857 C/T have been studied extensively to determine their association with the response to TNFα blockers in different SpA subgroups (including for AS, IBD/ CD, and PsA/Ps)^12^,^13^,^14^,^15^,^16^,^17^,^18^,^19^,^20^,^21^. However, these studies have yielded inconsistent findings, due in part to small sample sizes and inclusion of different patient populations. To provide further insight into the role of TNFα −308 A/G, −238 A/G, and −857 C/T polymorphisms in predicting treatment outcome of TNFα blockade in patients with SpA (PsA/Ps, AS, IBD/CD), we performed an intensive meta-analysis of published data and one set of unpublished data from our lab.

## Results

### Ten studies and one unpublished data set were used in the meta-analysis

**Figure 1:**
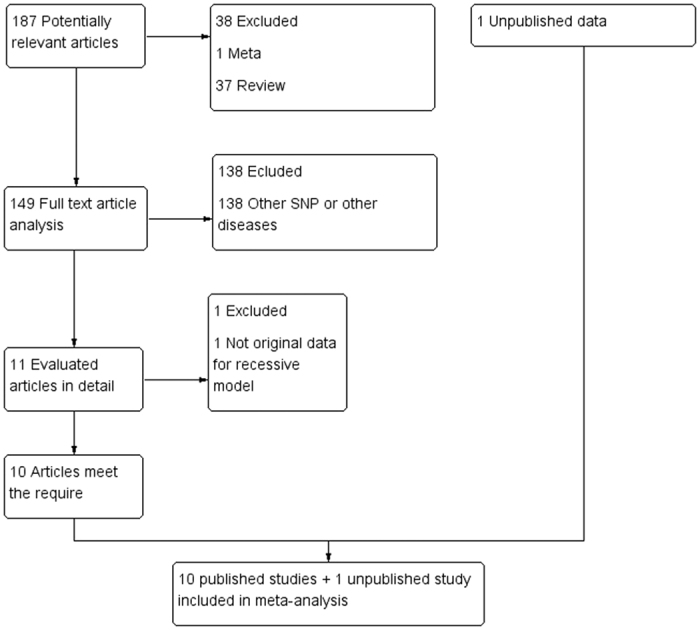
Flow Chart of the Selection Process for Analysis.

After filtering the studies for several criteria, we selected 10 articles^12^,^13^,^14^,^15^,^16^,^17^,^18^,^19^,^20^,^21^. Of the published studies, 3 were relevant to AS, 3 to CD/IBD, and 5 to Ps/PsA. A flow chart showing the selection process is shown in Fig. 1. In the meta-analysis, the selected articles contained 10 published studies and one unpublished study of responders and non-responders that allowed exploration of the association between the response to TNF blockers and TNFα −308 A/G, −238 A/G, and −857 C/T polymorphisms. The overall number of patients was 1,016 and the populations came from Europe (including Belgium and Switzerland) and China (Supplemental Table S1).

### Results of our own unpublished data

We assessed the association between the TNFα −308 A/G, TNFα −238 A/G polymorphisms and the response to TNF blockers according to the criteria of clinical responses. Because of the limited number of patients were included, all of the prediction did not get significant level and it was required to add to the meta analysis to get more persuasive conclusion. The detailed result was elucidated in Table S2. As for TNF −308, OR was 1.16 and 0.98 respectively for ASAS20 and ASAS40 and was 1.39, 1.02 respectively for BASDAI20 and BASDAI50. According to the result we could find that the result was quite dependent on the criteria response.

### Gender ratio, disease type, TNF blockers, and continent—sources of heterogeneity

Regression meta-analysis was performed to search for the source of heterogeneity in each article. Age, gender ratio, and disease duration were analyzed in only 8 of the 10 articles, because Lopez-Hernandez *et al*. and Giuseppe Murdaca *et al*. did not present this information. All other factors were analyzed in all 10 of the articles. Table 1 shows gender ratio was sufficient to explain heterogeneity. Disease type, continent, TNF blockers, and disease duration could explain approximately 70%, 53.7%, 41% and 16.86% of the heterogeneity, respectively. In SpA and AS patients, the association between TNF-α polymorphisms (−308 A/G, −238 A/G, −857 C/T) and response to TNF blockers was influenced by different diseases, TNF blockers, continent, disease duration, and gender ratio of selected populations but not by the patient number, follow-up period, or age of the selected population.

### Association between TNFα −308 A/G and −238 A/G polymorphisms and response to the TNF blockers

**Figure 2:**
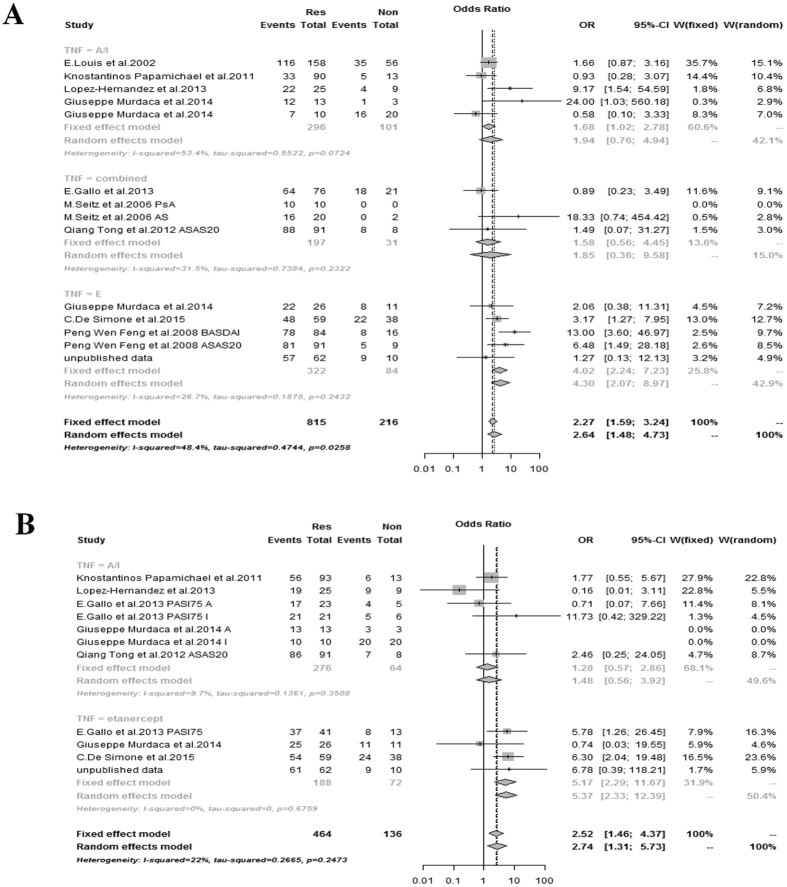
Odds ratios (ORs) and 95% confidence intervals (CI) from each study testing association of (**A**) −308 G > A TNFα polymorphism and (**B**) −238 G > A to the response to TNF blockers in different TNF blockers in SpA patients. If p-value < 0.1 we used the result of random effects model. Otherwise, a fixed effect model was used. A/I: adalimumab/infliximab, events: number of subjects with the common allele (G) observed, Res: responder, Non: nonresponder.

The meta-analysis of the selected studies showed an association between the TNFα −308 A/G polymorphism and the response to TNF blockers (OR = 2.64; 95% CI = [1.48–4.73]) (Fig. 2A). TNFα −238 A/G was also associated with the response to TNF blockers (OR = 2.52; 95% CI = [1.46–4.37]) (Fig. 2B). TNF blockers were organized into subgroups according to whether they were monoclonal antibodies (infliximab and adalimumab) or soluble receptors (etanercept), and analysis showed that each subgroup had a different OR. In the etanercept (Dimeric fusion protein) subgroup, associations between TNFα −308 GG or −238 GG polymorphisms and response to the TNF blockers were both significant (OR was 4.02 and 5.17, respectively) (Fig. 2A,B). However, in the infliximab/adalimumab (chimeric monoclonal antibody) subgroup, associations between TNFα −308 GG or −238 GG polymorphisms and response to TNF blockers were not significant. Overall, there was no heterogeneity in any of the subgroups except for in the infliximab/adalimumab (chimeric monoclonal antibody) subgroup for the −308 GG polymorphism (Fig. 2A,B).

### Association between the −857 C/T polymorphism and response to TNF blockers

**Figure 3:**
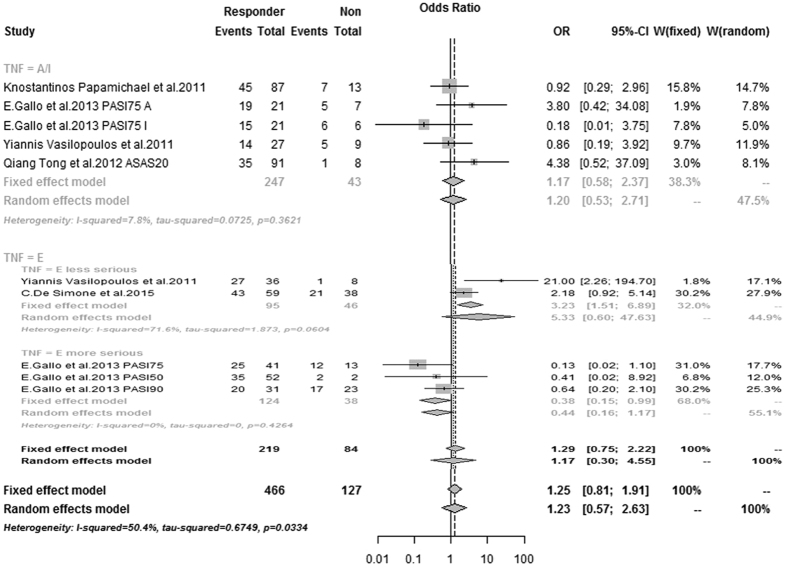
Odds ratios (ORs) and 95% confidence intervals (CI) from each study testing association of −857 C > T TNFα polymorphism and response to TNF blockers in SpA patients. If p-value < 0.1 we used the result of random effects model. Otherwise, a fixed effect model was used. A/I: adalimumab/infliximab, events: number of subjects with the common allele observed, Res: responder, Non: nonresponder.

The meta-analysis showed no association between the TNFα −857 TT polymorphism and the response to TNF blockers (OR = 1.23: 95% CI = [0.57–2.63]). However, marked heterogeneity existed in the etanercept subgroup (I^2^ = 70.9%, p = 0.0081), although no heterogeneity existed in the infliximab/adalimumab (chimeric monoclonal antibody) subgroup (I^2^ = 7.8%, p = 0.36). Based on these data, we performed further analyses according to the baseline condition of the patients in the etanercept subgroup. We defined longer mean disease duration and higher mean PASI (PASI > 20) as characteristics of a more serious disease condition. In patients with a more serious condition, those with the −857 TT polymorphism had a better response to etanercept (OR = 0.38: 95% CI = [0.15–0.99]), while in patients with a less serious condition, those with the −857 CC polymorphism had a better response to etanercept (OR = 3.23: 95% CI = [1.51–6.89]) (Fig. 3).

### Cumulative meta-analysis in AS patients

**Figure 4:**
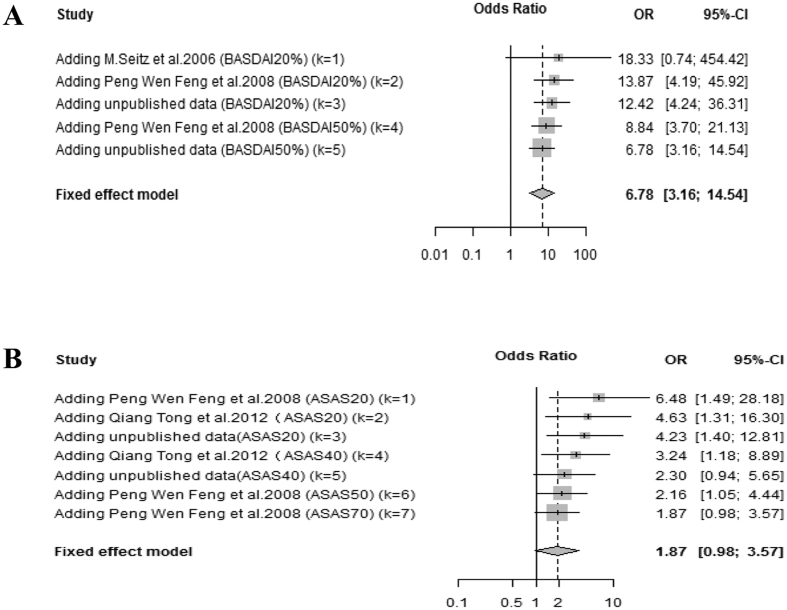
Cumulative meta-analysis of associations between −308 G− > A TNFα polymorphism and response to the TNF blockers in AS patients (**A**) BASDAI response criteria, (**B**) ASAS response criteria.

Cumulative meta-analysis was performed for 3 articles and our unpublished data to further investigate whether different criteria would influence the association between disease response and the polymorphisms. The selected data were all relevant to the different response criteria in the AS subgroup. Interestingly, OR decreased with stricter response criteria for both ASAS criteria (OR changed from 6.48 to 1.87) and BASDAI criteria (OR changed from 18.33 to 6.78) (Fig. 4). The overall result suggested that the response criteria were a source of heterogeneity in AS patients.

### No publication bias and sensitivity analysis

Publication bias was first examined qualitatively by funnel plots and estimated by Begg’s and Egger’s tests. The p-values were 0.29 (−308 GG), 0.25 (−238 GG), 0.75 (−857 CC) in Egger’s test and 0.41 (−308 GG), 0.32 (−238 GG), 0.79 (−857 CC) in Begg’s test. Based on these results, we concluded that there was no publication bias in our selected articles.

**Figure 5:**
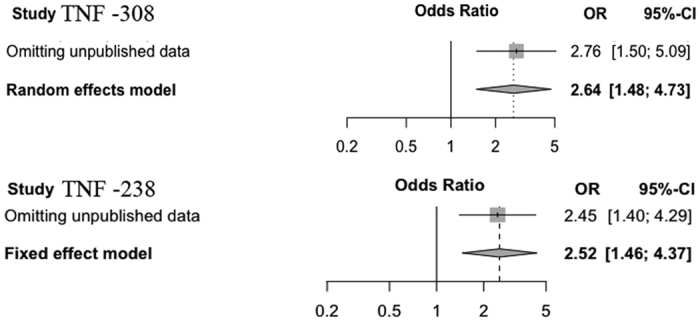
Sensitivity analysis of our own unpublished data.

In order to prove that our unpublished data could be integrated to the meta analysis, sensitivity analysis was performed. As described in Fig. 5, there was no obvious influence after omitting our own unpublished data.

## Discussion

Some of the studies included in this meta-analysis had identified associations between SpA patient response to TNF blockers and the presence of TNFα −308 A/G, −238 A/G, and −857 T/C polymorphisms, while others had not^12^,^13^,^14^,^15^,^16^,^17^,^18^,^19^,^20^,^21^. In our meta-analysis, a G allele at −308 or −238 predicted a good response to TNF blockers. A previous study found that subjects with an allele harboring the −308 A TNF promoter polymorphism produced elevated levels of TNF^22^. In that study, subjects with relatively high TNF levels tended to not respond to TNF blockers. The increased baseline expression of TNF in these patients may have prevented TNF blockers from decreasing TNF levels to below normal levels.

We found that TNFα polymorphisms could predict the response to etanercept (for TNF −238, OR = 2.52, CI = [1.46–4.37]) but not infliximab/adalimumab (for TNF −238, OR = 1.28, CI = [0.57–2.86]). Etanercept is a TNFR:Fc recombinant drug made up of two p75 receptors adjacent to the Fc portion of IgG1. Infliximab is an IgG1κ monoclonal antibody from mouse that neutralizes the biological activity of human TNFα^23^. A previous study determined that RA (rheumatoid arthritis) patients treated with infliximab for 6 weeks produced anti-infliximab antibodies to inhibit binding of the drug to TNFα. Additionally, levels of functional infliximab were significantly lower in patients with that antibody^24^. For AS patients treated with etanercept, levels of etanercept were not significantly different between responders and non-responders, suggesting that these patients did not produce antibodies to etanercept^25^. Overall, the percentage of patients who produced anti-drug antibody was 15–50% for infliximab and less than 10% for etanercept. Moreover, anti-etanercept antibodies had no influence on the efficacy of etanercept^26^. On the other hand, treatment with TNF blockers was likely to induce immunogenicity affecting mainly monoclonal antibodies such as infliximab^25^,^27^. Based on these findings, we postulate that the production of anti-infliximab antibody in patients treated with infliximab could be a potential confounding factor in using TNFα promoter polymorphisms to predict the response to TNF blockers.

In our cumulative meta-analysis of AS patients, we found that the association between the TNFα −308 A/G polymorphism and the response to TNF blockers was dependent on response criteria. Stricter BASDAI or ASAS criteria decreased OR. Interestingly, although OR decreased with stricter criteria, the 95% confidence interval (CI) became smaller, suggesting that the stricter response criteria was excluding unknown confounders (Fig. 4). This indicated that although TNFα −308 A/G was able to only weakly predict the response to TNF blockers under these stricter conditions, this result was much more convincing.

To find the heterogeneity in the selected articles, we performed meta-regression analysis. We concluded that TNF blockers, continents, disease duration, and gender ratio of selected populations were the source of heterogeneity. We also performed further analyses of different disease types. We found that the TNFα −308 A/G polymorphism could predict the response to TNF blockers in Ps patients and CD patients, but not in AS patients (Supplemental Fig. 1). That difference in the success of TNFa in predicting the response to TNF blockers may result from differences in the pathogenicities and genetic backgrounds of AS and CD/Ps. AS affects the axial skeleton and can lead to impaired spinal mobility. While TNF blockers have been shown to influence the inflammatory response in AS, there was no significant difference in the change in radiographic mSASSS in AS patients after treatment with etanercept, adalimumab, or infliximab for 2 years compared to baseline^28^,^29^,^30^. On the other hand, TNFα plays an important proinflammatory role in Ps patients. TNFα activates production of osteoclasts, promoting bone resorption and joint damage. Consistent with this role, TNFα blockers are effective in attenuating or halting the damaging pathophysiology of Ps^11^,^31^,^32^,^33^. In contrast, in AS, the relationship between TNFα and disease pathophysiology is not well known. On a genetic level, it has been reported that in PsA patients, TNFα-238, TNF-α308, and TNFα-857 have significant linkage disequilibrium (LD)^34^. However, analysis of our own unpublished data from AS patients showed the three SNPs did not have LD (r^2^ < 0.01, data not shown). These findings demonstrate the existence of heterogeneity among different types of Spondyloarthritis.

There are several important differences between the present study and two related meta-analyses that had been published previously^35^,^36^. In the present study, we included more patients than either of the two previous studies and added a set of unpublished data from our lab. In addition, unlike the two previous meta-analyses, we compared different TNF blockers. Finally, through the cumulative meta-analysis and meta-regression analysis, we proved that the linkage of the TNFα-238, TNF-α308, and TNFα-857 polymorphisms to treatment response to TNF blockers was dependent on the response criteria, disease type, continent, disease duration, and gender ratio. The results of our meta-analysis indicated that the common alleles of TNFα −238 and −308 could better predict the response to TNF blockers. These results are in agreement with those of previous reports in a meta-analysis conducted in SpA patients. However, our findings about whether TNFα −857 CC could predict the response to TNF blockers were different from those of a previous meta-analysis. In this study, we found that the TNFα −857 CC genotype could not predict a good response to adalimumab/infliximab, but it could predict the response of etanercept according to the baseline condition of the patients in this subgroup.

Some limitations of the meta-analysis should be taken into consideration. First, more subjects should be included to obtain a more powerful result. Second, in our own data, we concluded that response criteria was one of the sources of heterogeneity. We could not do subgroup analysis in other diseases because data was unavailable. In addition, although in the meta-regression analysis we knew that ethnicity was a source of heterogeneity, we were not able to perform further analysis because most of the selected data were from Caucasian patients and only three were from Chinese patients (including our unpublished data). Considering the confounding effect of response criteria, one article performed the response evaluation at a long term^37^, and got that TNF −308 A allele predicted better response to TNF blockers which was quite different from previous published results. So in the future, we would take a longer time of treatment to evaluate the response to TNF blockers. We will further analyze the association between TNFα polymorphisms and the response to TNF blockers in larger patients sizes after adjusting for the confounding factors mentioned in this meta-analysis.

## Methods

### Study selection and data extraction

We performed a search for studies that examined the associations between the responses of SpA patients to TNF blockers and the presence of TNFα −308 A/G, −238 A/G, or −857 C/T polymorphisms. The literature search was performed within the databases PubMed (Language: English) and CNKI (Language: Chinese) and included studies published in Nov 2015 or earlier. We searched for studies containing the following keywords in the databases: ‘tumor necrosis factor’ or ‘TNFα’; ‘polymorphism 308’, ‘polymorphism 238’, or ‘polymorphism 857’; ‘TNF blocker’, ‘TNF therapy’, ‘etanercept’, ‘infliximab’, or ‘adalimumab’; and ‘spondyloarthritis’, ‘SpA’, ‘ankylosing spondylitis’, ‘AS’, ‘psoriatic arthritis’, ‘PsA’, ‘Crohn’s disease’, or ‘CD’. All articles found using the above strategy were read thoroughly. Recessive modeling was performed in order to include most of the articles and obtain a large enough sample size.

Several additional criteria were used to select articles for this study:

1. It was an original article;
2. The study included at least one of the three SNPs of interest (TNFα −308 A/G, −238 A/G, or −857 C/T);
3. The article had to have determined genotype frequency.

Two investigators independently screened titles, abstracts, and full articles.

### Subjects, Genotyping and statistics analysis of our own unpublished data

The AS patients in our study were being treated at Guanghua Hospital and Changhai Hospital. This study was approved by the Ethical Committees of the School of Life Sciences of Fudan University and all participants provided written informed consent. AS was diagnosed according to 1984 modified New York criteria. All of the 72 patients were given subcutaneous injections of Etanercept 25 mg twice a week according to standard operating procedure and clinical information was collected at intervals of 0, 2, 4, 8, 12 weeks. In addition the biochemical indexes (including CRP (C-reactive protein)’ ESR (erythrocyte sedimentation rate), etc.), disease duration, HLA-B27 status, and so on. The disease activity of AS patients was evaluated using the Bath AS disease activity index (BASDAI) and criteria of the Assessment of SpondyloArthritis International Society (ASAS). Clinical response was evaluated according to ASAS^38^ improvement (ASAS20, ASAS40) and BASDAI^39^ improvement (BASDAI 20, BASDAI50) criteria at the end of 12 weeks.

Peripheral blood was collected from blood samples of the patients. Genomic DNA was then extracted form whole blood using a QIAamp DNA Blood Mini kit (QIAGEN, Germany) and stored at −20 °C immediately. The concentration and quality of isolated DNA was evaluated by a Nanodrop Lite spectrophotometer (Thermo Fisher’s Scientific, Waltham, MA, USA) according to the optical density (OD) 260/280 and 260/230 measurements. The experiment approaches were carried out in accordance with the approved guidelines.

Genotyping of TNF −238, −308 was performed by Sanger Sequencing and the primer was designed by ourselves. Patient genotypes were determined by screening all peak figures using CodonCode Aligner software. The statistical analysis of our unpublished data was calculated by Fisher’s exact test, and values below 0.05 was considered statistically significant.

### Statistical analysis

A meta-regression model was first used to explore sources of heterogeneity across studies^40^. Factors including sample size, gender, publication year, disease, continent, age, TNF blockers, and follow-up period were tested by meta-regression analysis in the recessive model. Each meta-regression analysis was justified for one factor. Factors with P* ≤ 0.1 and R^2^ > 5% were considered as sources of heterogeneity.

In order to further explore the sources of heterogeneity among studies, we stratified the extracted data according to the TNF blocker used. For studies with extreme heterogeneity (p < 0.01), extracted data was further stratified. Since different studies used distinct AS response criteria, such as ASAS20, ASAS40, BASDAI20%, BASDAI50%, cumulative meta-analysis was performed to identify whether results in AS patients were dependent on response criteria.

We assessed the Q statistic to determine the heterogeneity among studies^41^. P-values for heterogeneity over 0.1 indicated no heterogeneity among studies, and for this case we used a fixed-effect model with Mantel-Haenszel (M-H) method to calculate the pooled ORs^42^. When P-values for heterogeneity were less than 0.1, the DerSimonian and Laird method (D + L) random-effect model was utilized^43^.

Finally, through creating funnel plots qualitatively and as estimated by Begg’s^44^ and Egger’s test^45^, publication biases were assessed quantitatively.

Statistical analysis was performed by the ‘*meta*’ package in R (Version 3.2.2: www.r-project.org/). P-values < 0.05 in two-tailed tests were regarded as statistically significant.

## Additional Information

**How to cite this article**: Liu, J. *et al*. TNF-α Promoter Polymorphisms Predict the Response to Etanercept More Powerfully than that to Infliximab/Adalimumab in Spondyloarthritis. *Sci. Rep.* **6**, 32202; doi: 10.1038/srep32202 (2016).

## Supplementary Material

Supplementary Information

## Figures and Tables

**Table 1:** Meta-regression analysis in the meta-analysis.

Log(OR)	β	t	P > |t|	95%	CI	I^2^(%)	τ^2^	R^2^(%)
	10 articles							
Year§	−0.03	−0.39	0.69	−0.19	0.12	56.00	0.78	0
**Disease**	−1.51	−2.60	0.009	−2.64	−0.37	24.15	0.16	**70.01**
**TNF Blockers**	−1.01	−1.81	0.07	−2.10	0.08	36.43	0.31	**41.46**
**Continent**	−1.33	−2.10	0.04	−2.58	−0.08	32.92	1.49	**53.70**
Followup Period§	−0.03	−0.61	0.54	−0.12	0.06	56.05	0.68	0
Patient Number§	−0.005	−0.910	0.360	−0.020	0.006	51.39	0.62	0
**Overall Above**						17.80	1.22	**72.44**
	8 articles							
Age§	−0.03	−0.94	0.35	−0.11	0.04	60.41	0.64	0
**Gender(male/female**§	0.17	3.37	0.0007	0.07	0.27	0.00	0.00	**100**
**Disease Duration**§	−0.11	−1.30	0.19	−0.26	0.05	57.31	0.60	**16.86**

^§^These factors were treated as continuous data. Sources of heterogeneity are bolded.
