# Supplementary material for: TNF-α Promoter Polymorphisms Predict the Response to Etanercept More Powerfully than that to Infliximab/Adalimumab in Spondyloarthritis
Source: Sci Rep. 2016 Aug 31;6:32202. doi: 10.1038/srep32202 (PMC5006048; doi:10.1038/srep32202)
Supplement: Supplementary Information [file srep32202-s1.doc]

**TNF-α Promoter Polymorphisms Predict the Response to Etanercept More Powerfully than that to Infliximab/Adalimumab in Spondyloarthritis**

Jing Liu^1^, Zheng Dong^1^, Qi Zhu^2^, Dongyi He^2^, Yanyun Ma^1^, Aiping Du^1^, Fan He^1^, Dongbao Zhao^3^, Xia Xu^3^, Hui Zhang^1^, Li jin^1^, Jiucun Wang^1,4*^

^1^State Key Laboratory of Genetic Engineering and Ministry of Education (MOE) Key Laboratory of Contemporary Anthropology, School of Life Sciences, Fudan University, Shanghai, China

^2^Guanghua Integrative Medicine Hospital, Shanghai, China; Institute of Arthritis Research, Shanghai Academy of Chinese Medical Sciences, Shanghai, China

^3^Department of Rheumatology and Immunology, Changhai Hospital, affiliated to second military medical university, Shanghai, China

^4^Institute of Rheumatology, Immunology and Allergy, Fudan University, Shanghai, China

* Corresponding author. Correspondence and requests for materials should be addressed to J. W. (email: jcwang@fudan.edu.cn)

**Supplemental Table 1.** Characteristics of 10 previous published articles and one unpulished data of our group

| Study | Year | Country | Disease | TNF blocker | patient number | Criteria | follow up (weeks) | site | Reference |
| --- | --- | --- | --- | --- | --- | --- | --- | --- | --- |
| E.Louis *et al.* | 2002 | Belgium | CD | Infliximab | 214 | CDAI | 4 | -308 | [12] |
| M.Seitz *et al.* | 2006 | Switerzland | AS/PsA | Combined | 22 | BASDAI | 24 | -308 | [13] |
| Peng Wen Feng *et al.* | 2008 | China | AS | Etanercept | 100 | BASDAI/ASAS | 12 | -308 | [14] |
| Knostantinos Papamichael *et al.* | 2011 | Greek | CD | Infliximab | 106 | HBI | 12 | -238,-308,-857 | [15] |
| Yiannis Vasilopoulos *et al.* | 2011 | PsA | PsA | Etanercept Adalimumab/Infliximab | 80 | PASI | 24 | -857 | [16] |
| Qiang Tong *et al.* | 2012 | China | AS | Infliximab/rhTNFR-Fc | 99 | ASAS | 12 | -238,-308,-857 | [17] |
| Lopez-Hernandez *et al.* | 2013 | Spain | IBD | Adalimumab/Infliximab | 34 | HBI | 4 | -238,-308 | [18] |
| E.Gallo *et al.* | 2013 | Spain | Ps | Etanercept Adalimumab Infliximab | 109 | PASI | 24 | -238,-308,-857 | [19] |
| Giuseppe Murdaca *et al.* | 2014 | Italy | PsA | Etanercept Adalimumab Infliximab | 83 | DAS28 | 24 | -238,-308 | [20] |
| C.De Simone *et al.* | 2015 | Italy | Ps | Etanercept | 97 | PASI | 12 | -238,-308,-857 | [21] |
| Unpublished data | 2015 | China | AS | Etanercept | 72 | BASDAI/ASAS | 12 | -238,-308 |  |
| BASDAI: Bath Ankylosing Spondylitis Disease Activity Index; AS:ankylosing Spondylitis; ASAS: ASsessment in Ankylosing Spondylitis international Society CD: Crohn’s disease; CDAI: Crohn’s Disease Activity Index; DAS28: Disease activity score 28; HBI: Harvey–Bradsaw Index;  Ps: Psoriasis; PASI: Psoriasis Area and Severity Index; PsA: Psoriatic arthritis; rhTNFR-Fc: TNF-α receptor II–IgG Fc fusion protein. | | | | | | | | | |
|
|  |  |  |  |  |  |  |  |  |  |
|  |  |  |  |  |  |  |  |  |  |

**Supplemental Table 2. The result of the statitics analysis on our own unpublished data**

|  |  | response | | non-response | | OR | 95% CI |
| --- | --- | --- | --- | --- | --- | --- | --- |
| Gene site | creteria response | GG | GG+AG | GG | GG+AG |  |  |
| -308 | ASAS20 | 62 | 67 | 4 | 5 | 1.16 | 0.23-6.09 |
|  | ASAS40 | 52 | 57 | 14 | 15 | 0.98 | 0.40-2.42 |
|  | BASDAI20 | 64 | 69 | 2 | 3 | 1.39 | 0.15-17.11 |
|  | BASDAI50 | 57 | 62 | 9 | 10 | 1.02 | 0.35-3.07 |
| -238 | ASAS20 | 65 | 67 | 5 | 5 | 0.97 | 0.21-4.43 |
|  | ASAS40 | 56 | 57 | 14 | 15 | 1.05 | 0.43-2.60 |
|  | BASDAI20 | 67 | 69 | 0 | 3 | / | 0.38-/ |
|  | BASDAI50 | 61 | 62 | 9 | 10 | 1.09 | 0.37-3.27 |


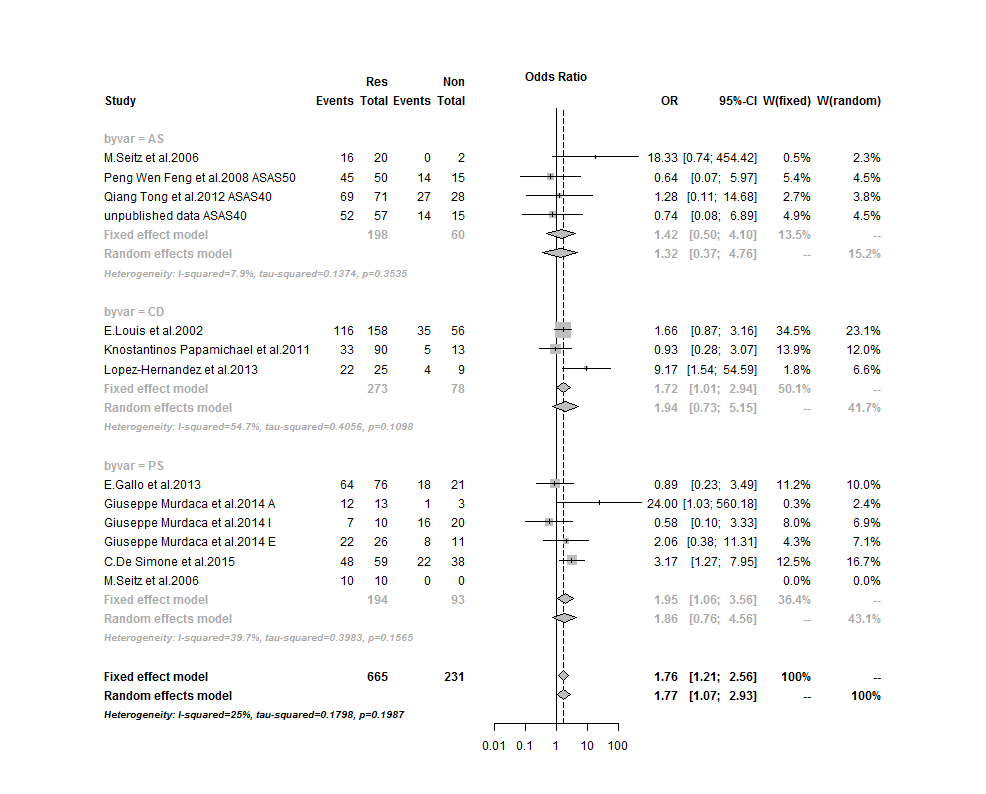
 **Supplemental Figure 1** Odds ratios (ORs) and 95% confidence intervals (CI) from each study testing association of-308G>A TNFα polymorphism to the TNF blockers in different disease types. If P-value<0.1 we used the result of random effects model, otherwise, fixed effect model was performed. A/I: adalimumab/infliximab， events: number of subjects with the common allele observed, Res: responder, Non: nonresponder
